# Lean Body Mass and Muscle Cross-Sectional Area Adaptations Among College Age Males with Different Strength Levels across 11 Weeks of Block Periodized Programmed Resistance Training

**DOI:** 10.3390/ijerph18094735

**Published:** 2021-04-29

**Authors:** Paul A. Moquin, Alexander B. Wetmore, Kevin M. Carroll, Andrew C. Fry, W. Guy Hornsby, Michael H. Stone

**Affiliations:** 1 Center of Excellence for Sport Science and Coach Education, Department of Sport, Exercise, Recreation and Kinesiology, East Tennessee State University, Johnson City, TN 37614, USA; awetmore425@gmail.com (A.B.W.); carrollk@etsu.edu (K.M.C.); stonem@etsu.edu (M.H.S.); 2 Osness Human Performance Laboratories, Department of Health, Sport and Exercise Sciences, University of Kansas, Lawrence, KS 66045, USA; acfry@ku.edu; 3 College of Physical Activity and Sport Sciences, West Virginia University, Morgantown, WV 26505, USA; William.hornsby@mail.wvu.edu

**Keywords:** block periodization, lean body mass, total body water, strength, cross-sectional area

## Abstract

The block periodization training paradigm has been shown to produce enhanced gains in strength and power. The purpose of this study is to assess resistance training induced alterations in lean body mass and cross-sectional area using a block periodization training model among individuals (n = 15) of three differing strength levels (high, moderate and low) based on one repetition maximum back squat relative to body weight. A 3 × 5 mixed-design ANOVA was used to examine within-and between-subject changes in cross-sectional area (CSA), lean body mass (LBM), lean body mass adjusted (LBM_adjusted_) and total body water (TBW) over an 11-week resistance training program. LBM_adjusted_ is total body water subtracted from lean body mass. The ANOVA revealed no statistically significant between-group differences in any independent variable (*p* > 0.05). Within-group effects showed statistically significant increases in cross-sectional area (*p* < 0.001), lean body mass (*p* < 0.001), lean body mass adjusted (*p* ˂ 0.001) and total body water (*p* < 0.001) from baseline to post intervention: CSA: 32.7 cm^2^ ± 8.6; 36.3 cm^2^ ± 7.2, LBM: 68.0 kg ± 9.5; 70.6 kg ± 9.4, LBM_adjusted_: 20.4 kg ± 3.1; 21.0 kg ± 3.3 and TBW: 49.8 kg ± 6.9; 51.7 kg ± 6.9. In conclusion, the results of this study suggest subjects experienced an increase in both lean body mass and total body water, regardless of strength level, over the course of the 11-week block periodized program. Gains in lean body mass and cross-sectional area may be due to edema at the early onset of training.

## 1. Introduction

Theoretical considerations indicate that for optimum enhancement of maximum strength and power, initial training should emphasize body composition alterations and enhancement of metabolic/work capacity [1,2,3,4,5,6]. Evidence indicates that alterations in body composition, gains in lean body mass (LBM) and loss of fat are better accomplished using higher volumes of resistance training [5,7,8,9]. LBM largely consists of muscle, connective tissue and bone. Although resistance training can affect alterations in all of these constituents, increase in measured LBM is largely due to muscle hypertrophy [10].

Several factors likely affect the degree to which hypertrophy impacts strength and power development. These include the type of hypertrophy and the initial strength/LBM values. Hypertrophy potentially takes two forms, sarcoplasmic hypertrophy (SH) and myofibrillar hypertrophy (MH) [11,12,13]. Characteristics of SH include increases in sarcoplasmic proteins, glycogen and/or sarcoplasm; whereas MH is characterized by an increase in contractile proteins [13]. Recent evidence indicates initial hypertrophy may be largely sarcoplasmic in nature and depends upon a large influx of fluid (edema) in response to damage and inflammation [14,15,16]. 

Although there can be individual variation [11], meaningful contractile related hypertrophy likely does not occur for several weeks after training is initiated [14,15]. During the onset of training, the impact on strength and power can be relatively small compared to other factors such as neurological adaptations, tissue stiffness, etc.; however, reviews of the literature indicate MH, resulting from long-term resistance training, does appear to substantially contribute to strength development [17,18].

There is evidence from both early muscle activation and cross-sectional area (CSA) studies [19,20] and later CSA studies [14,15] indicating that initial gains (first 6–8 weeks) in MH are negligible to small and likely do not contribute markedly to increased strength and power. This evidence also suggests that later (after ≈ 8 wks) alterations in MH can begin to contribute to alterations in strength, power and related characteristics. 

Indirect evidence suggests it is also possible consistent bodybuilding type resistance training (high repetitions per set, training to failure) may result in greater SH [11,21]. Perhaps this hypertrophic difference partially explains observations indicating bodybuilders are not as strong or as powerful as other strength-power athletes in multi-joint absolute [22] relative [23] or single fiber [21] measures. 

Past studies have shown evidence as to why initial resistance trained increases in LBM and muscle CSA do not always associate with gains in strength and related characteristics, particularly among untrained and minimally trained subjects. One such study indicates that initial maximum strength levels and initial CSA can influence subsequent adaptation in CSA and LBM [17]. Certain resistance training programs, particularly those using periodization programming, alter several factors over time including volume and intensity. Alterations in programming appear to produce enhanced gains in strength and power and, perhaps, muscle CSA [1,2,17,24]

It is not clear to what extent training program alterations in resistance training volume and intensity impact alterations in muscle CSA and LBM. Additionally, it is not clear as to the impact of initial maximum strength levels, muscle CSA and LBM on alterations in muscle CSA and LBM. 

The purpose of this study was to assess the degree of resistance training induced alterations in CSA and LBM by examining the effect of (1) the training, and (2) initial strength levels and body composition with an emphasis on muscle mass.

## 2. Materials and Methods

### 2.1. Subjects

Based on results of previous investigations [25], power analysis for repeated measures, between factors ANOVA with a moderate effect size was calculated (alpha = 0.05, f = 0.9, number of groups = 3, number of measurements = 5). It was determined that a sampled size of 15 was needed (Franz Faul, Kiel, Germany, Gpower vers. 3.1.9.2). Fifteen males of varying strength levels volunteered to participate and completed the study (age = 24.1 ± 3.4 yrs, body mass = 89.1 ± 17.0 kg, BMI = 28.2 ± 5.3). 

Correlations indicate a strong and consistent negative relationship between the initial 1-RM and gains in performance. This would indicate that weaker subject’s progress at a greater rate than stronger subjects. Considering these correlations, subjects were divided into three strength groups (High, Moderate and Low) based upon their initial 1-RM back squat in accordance with the findings of Suchomel et al. [26]. 

Subjects did vary in terms of both training age and training experience. Those who had more experience with block periodization or resistance training should have a greater initial relative strength compared to novice or inexperienced lifters overall. The subject’s success in the back squat was not influenced by the knowledge of the test but more so by the overall strength that comes with experience in resistance training. This is very similar to a collegiate weight room with upperclassmen and incoming freshmen. 

Subjects (n = 7) unable to back squat at least 1.25 kg/kg were considered low (age = 23.3 ± 4.3 yrs, BM = 83.5 ± 18.5 kg, BMI = 26.5 ± 5.3). Subjects able to back squat between 1.25–1.75 kg/kg were considered moderate (n = 4) (age = 25.3 ± 3.2 yrs, BM = 100.2 ± 17.9 kg, BMI = 31.0 ± 5.6). Subjects able to back squat greater than 1.75 kg/kg were considered high (n = 4) (age = 24.3 ± 2.2 yrs, BM = 87.7 ± 10.0 kg, BMI = 28.2 ± 4.9). 

The groups were separated based on findings of Suchomel et al. [26]. Suchomel et al. found subjects were considered strong if able to squat 2× body weight and weak if they were unable to squat at least 1.25× body weight. We elected to lower the requirements to 1.75× body weight. This value fits into the Strength Association Phase as out lined by Suchomel et al. and represents a “stronger” group of athletes/subjects. In order to find enough subjects to qualify for this study as having higher strength levels we chose to use the 1.75× value.

All subjects read and signed an informed consent document prior to participating in the study, as approved by the university’s Institutional Review Board.

### 2.2. Dietary Food Logs

Subjects were asked to fill out a 3-day dietary food log during the end of each training block. They were asked to maintain their regular diet and to continue using any supplements/medications in use the month prior to the start of the study. Food logs were analyzed for total kilocalorie intake and macronutrient intake (carbohydrates, proteins and fats) using Nutritionist Pro Diet Analysis Software (Axxya Systems, Stafford, TX, USA).

### 2.3. Procedures

A block periodization design was used for the resistance training program as it has been previously shown to be effective in developing maximum strength and power [27,28,29,30]. All subject completed four bioimpedance spectroscopy (BIS) baseline measurements, two ultrasound (US) measurements, one strength baseline measurement (1-RM back squat) and a testing session following each of the four training blocks. Subjects in the low strength group underwent a two-week familiarization phase to help minimize the learning effect from the back squat testing. Pre-testing took place one week before the beginning of the training intervention. Post-block testing sessions were completed three days following the last training session of the previous block and the start of the upcoming training block. All testing sessions were completed at the same time of day in the same order of tests. 

All groups completed the same non-failure strength training program and testing scheme. The training program followed a single factor block periodization model and was programmed with an emphasis on strength and power development.

The subjects completed three resistance training sessions per week (Monday, Wednesday and Friday) and two sprint sessions per week (Tuesday and Thursday) each week. The resistance training sessions were monitored by two NCAA strength and conditioning coaches who were certified strength and conditioning specialists (NSCA). These sessions were held in either the East Tennessee University weight room or the East Tennessee University Sport Science Laboratory weight room. The sprint sessions were monitored by the same two strength and conditioning coaches on an indoor track inside the East Tennessee University Mountain State Health Alliance Athletic Center. Sprints were performed to 100% intensity. The sprints consisted of 3 sets of 2 repetition 20 m sprints with 2 min of rest between repetitions and 4 min of rest between sets in accordance with Carroll et al. [25].

The groups followed a three phased programming emphasis (strength-endurance, maximal strength and power). This progression included a three-week taper at the end of the last block following a functional overreach. Heavy and light intensity days were included each week to both manage fatigue and ensure a spectrum of power outputs. Lastly, all training loads were selected using relative intensities (% set-rep best) [25]. The training program is shown in Table 1 (based on Carroll et al.) [25]. 

All lifts included the eccentric and concentric portion of the lift. The exercise selection is shown in Table 2. 

Prior to all BIS, US testing and strength testing (relative and absolute) subjects provided a urine sample to estimate their hydration level. Hydration was tested using a refractometer (Atago, Tokyo, Japan). Dehydration has been shown to have a negative effect on performance, cognitive abilities and ultimately testing results [31]. Subjects were required to have a USG < 1.020 before testing could begin.

An SFB7 BIS device (ImpediMed Limited, Pinkenba, Australia) was used to measure total body water (TBW) according to the methods used by Moon et al. [32]. Two readings were averaged for the measurement of total body water (TBW). 

**Figure 1:**
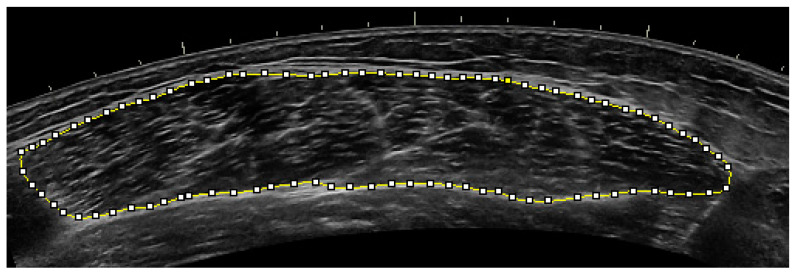
Cross-sectional area measurement.

A 7.5 MHz ultrasound (US) probe (LOGIQ P6, General Electric Healthcare, Wauwatosa, WI, USA) was used to measure cross-sectional area (CSA) of the vastus lateralis (VL). Two CSA images were attained using a panoramic image sweep perpendicular to the VL from the mid-point of the femur while the subject was standing and the measured leg unweighted to better reflect the functional architecture of the muscle in sporting activities [33]. Then, CSA was analyzed by selecting the best image that displayed the VL and using an image processing software (ImageJ 1.52a, National Institutes of Health, Bethesda, MD, USA) to trace the intermuscular area as shown in Figure 1. Each image was calibrated via converting millimeters to pixels. Once the calibration occurred and the image was traced, the software allowed the researcher to ‘spline’ the entire drawing and outline the muscle belly precisely with each individual dot. The ultrasound technician and researcher analyzing the data remained the same throughout the entire study. 

For dynamic strength, the subjects performed a 1-RM test for the back squat. Prior to the lift, a standardized warm-up was performed [33]. The warm up procedure is shown in Table 3.

The testing percentages were based on a subject’s estimated 1-RM and the trial-and-error method for the untrained subjects [34]. If the projected 1-RM was successful, the subject continued to attempt progressively heavier loads until a true 1-RM was reached. The back squat was deemed acceptable if the participant was able to squat to parallel (123.70 ± 2.03°) (determined by a line from the top of the knee to the hip-crease) with the floor or below [35]. Squat depth was determined by two experienced certified strength and conditioning specialists.

An equation was created in an attempt to investigate the potential difference between sarcoplasmic hypertrophy (edema) and myofibrillar hypertrophy (contractile elements), Equation (1). The TBW of each subject was subtracted from LBM. Adipose tissue consists of approximately 10% water and therefore, the total fat mass of the subject was multiplied by 0.1 [36]. This product was subtracted from the subject’s TBW prior to calculating the subject’s LBM adjusted for water in fat, Equation (2).

Equation (1). Lean body mass adjusted for water content

LBM_adjusted_ (kg) = LBM (kg) − TBW_adjusted_ (kg) (1)

Equation (2). Total body water adjusted for water in fat

TBW_adjusted_ (kg) = TBW (kg) − [(Body mass (kg) × percent body fat) × 0.1] (2)

Post block testing consisted of hydration, BIS, and US measures. Post block testing was completed after the 3-week strength endurance (SE) block, the 4-week maximum strength (MS) block, the 1-week functional overreach (FOR) and the 3-week taper. Post block testing was completed the Monday following the previous block’s last training session and prior to the next block’s training session that evening. 

**Figure 2:**
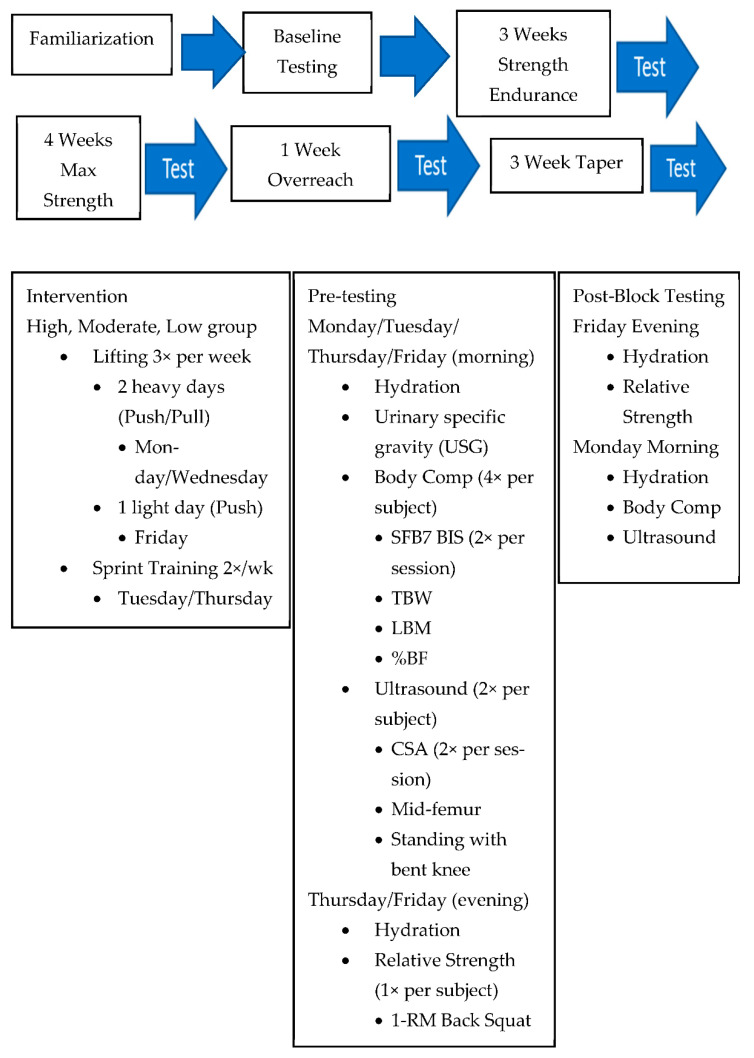
Research testing procedures.

The testing procedures for the research project is shown in Figure 2.

## 3. Statistical Analysis

All data have been expressed as mean ± standard deviation. 6 separate 3 × 5 mixed-design ANOVAs (group x time) were conducted for this study with an alpha level of *p* ≤ 0.05. Tests for homogeneity of variance (Levene’s Test) and Mauchly test of sphericity were calculated prior to performing ANOVA tests. If sphericity was violated, the Greenhouse-Geisser correction was used. The alpha level was set at *p* ≤ 0.05. Significant main effects were followed by post hoc tests using Holm-Bonferroni adjustment. Magnitudes of effect were calculated using Cohen’s *d* effect sizes. All statistics were conducted using JASP (JASP version 0.10.1). Cohen’s *d* magnitude thresholds are as follows: trivial (0–0.2), small (0.2–0.6), moderate (0.6–1.2), large (1.2–2.0) and very large (>2.0) [37]. 

Intraclass correlation (ICC) and coefficient of variation (CV) were calculated to analyze reliability of all US and BIS measures were performed using Microsoft Excel and SPSS 26.0 (IBM Corp., Armonk, NY, USA). The data below shows a high degree of reliability for all US and BIS measures. Reliability statistics are shown in Table 4. Supplemental data is provided on publication’s website.

## 4. Results 

### 4.1. Food Logs ANOVA

The ANOVA did not reveal a statistically significant interaction effect on total caloric intake (*p* = 0.39) or protein intake (*p* = 0.55). No statistically significant between-subject differences were observed in caloric intake (*p* = 1.00) or protein intake (*p* = 0.52) over the 11-wk intervention.

### 4.2. Bioelectrical Impedance Measures ANOVA

All dependent variables met the assumptions of normality and sphericity at the level of significance. Each variable was analyzed using a mixed (repeated measures ANOVA and between subject ANOVA). If there was a significant interaction effect, a Holm-Bonferroni Adjust post hoc analysis was calculated. If there was not a significant interaction effect, such was the case for all between-subject (high, moderate, low groups) variables at all time point and all measures of %BF, a post hoc analysis was not calculated. The ANOVA revealed a statistically significant interaction effect for TBW (*p* ˂ 0.001), LBM (*p* ˂ 0.001) and LBM_adjusted_ (*p* ˂ 0.03). The ANOVA did not indicate a statistically significant interaction effect for body mass (*p* = 0.28) or (percent body fat (*p* = 0.30) (Table 5). The ANOVA did not show any statistically significant differences for the between-subject interaction effect but did reveal very large effect sizes for TBW (*p* = 0.07; *d* = 1.67, [CI = 0.80–2.45]), LBM (*p* = 0.07, *d* = 1.67, [CI = 0.80–2.45]) and LBM_adjusted_ (*p* = 0.09, *d* = 1.59, [CI = 0.73–2.36]). Overall, from baseline to taper there was a statistically significant very large increase in TBW (*p* < 0.01; *d* = 1.37, [CI = 0.54–2.12]), LBM (*p* < 0.01; *d* = 1.37, [CI = 0.54–2.12]), LBM_adjusted_ (*p* < 0.01; *d* = 1.22, [CI = 0.41–1.97]) and a statistically significant moderate increase in BM (*p* = 0.03; *d* = 0.81, [CI = 0.04–1.53]). Results for change in BM, TBW, LBM and LBM_adjusted_ after each block for each group and the subject pool overall are shown in Table 5.

### 4.3. Ultrasonography Measures ANOVA

The ANOVA revealed a statistically significant interaction effect for CSA (*p* < 0.01) (Table 6). The ANOVA did not reveal any statistically significant differences between-subject effects of time but did show a large effect size for CSA (*p* = 0.14; *d* = 1.26, [CI = 0.45–2.01]). Overall, from baseline to taper there was a statistically significant large increase in CSA (*p* < 0.01; *d* = 1.22, [CI = 0.41–1.96]).

## 5. Discussion

Over the course of this 11-week intervention, there was no statistically significant difference between the three groups (high, moderate, and low) in terms lean body mass and CSA adaptions through block periodized training at any time point. This study also examined within-subject differences in terms of body composition and CSA and found all subjects increased to statistical significance from pre-to-post intervention. 

TBW adjustments were made to delineate the composition of alterations more clearly in LBM. To investigate this delineation, an assumption that the resulting LBM_adjusted_ reflects primarily protein alterations was made.

The results indicate LBM_adjusted_ increased over time (*p* < 0.03; *d* = 1.22), even with a statistically significant increase in TBW (*p* < 0.001; *d* = 1.37). The increase in TBW is at least partially explained by fluid retention in muscle, perhaps resulting from damage [15,38,39]. The greatest increase in TBW and LBM_adjusted_ occurred during the initial high-volume phase (baseline to SE). A similar trend for CSA (n = 15) was also noted. This observation may simply result from a relatively novel stimulus causing more damage and edema than occurred later in the program. Damas et al. [14] found similar results, increases in CSA and CSA echo intensity, following the first three weeks of a high-volume resistance training intervention among untrained subjects. Following their 10-week intervention, Damas et al. [14] found a statistical increase in CSA but not a statistical difference in CSA echo intensity; inferring initial muscular swelling at the initiation of high-volume resistance training (sarcoplasmic hypertrophy). The findings presented in this experiment support early edema followed by sustained muscular growth throughout the training program.

However, this trend of an initial large alteration in LBM_adjusted_ occurred regardless of strength level or training background. This observation suggests that a higher volume of training stimulates protein accretion to a greater extent than lower volume. Additionally, a drop in training volume, especially FOR to Taper, showed a loss of LBM_adjusted_. Suggesting training volume has a marked effect on protein accretion and maintenance. These observations agree with previous indications of the effect of volume on muscle hypertrophy [39,40]. However, the exact makeup of the protein accretion cannot be ascertained using the methods of this study (BIS and ultrasound). 

Interestingly, the presence of increased TBW is not necessarily detrimental to muscle performance. While increased TBW at the beginning of a resistance training program could mean edema and muscle damage, increased muscle fluid content can theoretically improve muscle force production. Fluid pressure within muscle acts as an intermediary between contractile proteins and extracellular matrix elements throughout the tissue [41]. An alteration of muscle internal fluid pressure could alter contractile force. Sleboda and Roberts [41] present evidence that increased intra and inter fiber fluid could enhance force transmission and potentially produce more contractile force through an increase in force transmitted to the extracellular matrix. Increased TBW could potentially improve performance of the muscle. This could partially explain, along with the nervous system, an initial increase in maximum strength with little indication of MH occurring. Regardless, the net effect of the training program was an increased LBM_adjusted_ over the 11-week intervention by approximately 0.7 kg. 

Although not statistically significant, it is important to note that, reflected by large effect sizes, initial levels of LBM, LBM_adjusted_ and maximum strength levels did appear to influence the gains in LBM and LBM_adjusted_ [42]. For example, LBM_adjusted_ showed a greater net improvement (baseline to taper) for the low group than both the high and moderate groups: High = 0.4 kg (2.1%), *d* = 0.79, (CI = −0.74–2.11); Moderate 0.7 kg (3.0%), *d* = 0.79, (CI = −0.74–2.11) and Low 0.8 kg (4.4%), *d* = 2.16, (CI = 0.72–3.29).

Lastly, while the low strength group consisted of mostly untrained subjects (7 untrained and 1 trained), the moderate and strong group each consisted of four trained subjects (based on resistance training for at least the past 12 months). In terms of body composition, the moderate group had higher pre-intervention levels of both LBM (moderate = 74.3 kg; strong = 70.6 kg) and LBM_adjusted_ (moderate = 22.5 kg; strong = 20.7 kg) than the strong group; however, the strong group had a higher percentage of LBM_adjusted_ compared to BM (strong = 23.6%; moderate = 22.5%) pre-intervention.

Although, it is well known that heredity influences physical and performance characteristics [28], it is also well-known resistance training influences these factors [43]. Further research will be needed to determine to what degree each of these factors (heredity versus previous training) affect training induced alterations. Regardless initial strength levels affect the adaptations.

### Limitations

One of the limitations of the current study is the limited sample size. To better infer the effect of training status on adaptions to training, further research and greater sample sizes is necessary.

A second limitation of the current study is the relatively short duration of 11 weeks (one stage). While the current study is one of the longest-term studies currently available, the ability to program and test subjects over multiple stages could be very informative as to how subjects adapt based on previous training status.

A third limitation of the current study was the inability to directly measure muscle tissue through an alternate means such as MRI, DEXA or muscle biopsy. These types of measure would allow a more direct assessment of muscle mass, and comparison of these measures to BIS would result in a better understanding of adaptions over the 11-week intervention. Unfortunately, these types of measurements are quite cost prohibitive for most laboratories but should be pursued in the future. Therefore, the investigators reasonably hypothesized that alterations in SH and MH would be reflected in measures of LBM and body water alterations using BIS.

Lastly, training history was not taken into consideration for this study. The purpose of the study was to assess potential alterations largely independent of training history. High and moderate strength subjects were grouped as such due to relative strength. While training history can be an important factor in training progress, it cannot easily be separated from maximum strength levels. Subjects in the moderate group were trained based on at least 12 months of resistance training experience. Furthermore, while the individual may exist, the authors with over 100 years of collective experience, have never observed an untrained subject that has been able to squat ≥1.75 kg/kg. To aid in minimizing a training/learning effect, a familiarization phase was undertaken. Untrained subjects (7 low strength subjects) participated in a familiarization phase 4 and 3 weeks prior to baseline testing. Proper technique was taught twice a week at this time using only the bar (20 kg) for 3 sets of 5 repetitions. During the same time period, the high and moderate strength subjects performed low volume training (≤5 repetitions and ≤3 sets) with constant loading (≈70–75% of 1 RM) (for four weeks) prior to baseline testing. It should be noted that in this study grouping the subjects as trained or untrained would not have obviated the result—the weaker, less trained subjects would still have progressed at a faster rate.

## 6. Conclusions

There was no statistically significant difference between the three groups in terms of body composition and CSA adaptions through 11 weeks of block periodized training. The data did indicate that all subjects increased to statistical significance from pre-to-post intervention in terms of body composition and CSA.

Potential sarcoplasmic/edema-based hypertrophy at the onset of a RT program and a continued alteration in LBM and CSA with drop in volume should be pursued, particularly with very well-trained subjects. If this pattern holds true for athletes, an increase in muscle edema with an increase in RT volume might lead to adverse effects in performance if introduced at the wrong point in time.

The results of this study suggest that subjects’ initial strength and LBM level can influence the gain in LBM and LBM_adjusted_ through RT and likely play a role in maximum strength (1-RM) alterations [44]. While subjects experienced an increase in hypertrophy after the introduction of RT, there should be consideration for the possibility of edema occurring in muscle. True myofibrillar hypertrophy may not occur until several weeks after the start of a new RT program. In conclusion, hypertrophy should be monitored not only through CSA measures but also using TBW measures. By only monitoring LBM or CSA, the researcher (and coaches) may be misled as to what is actually occurring in terms of protein accretion.

Future research topics include examining BIS and US measures alongside muscle biopsies. By doing so, practitioners may be able to track muscle edema in a much quicker and more cost-effective manner than muscle biopsy. As a practical application, strength and conditioning coaches who track body composition or lean body mass of their athletes should track total body water as well. It does not matter the athlete’s initial strength or lean body mass, according to this research, an increase in volume may lead to an increase in TBW. This may be attributed to edema and can be harmful to athletic performance. Strength and conditioning coaches should also consider where to program an increase in volume throughout their annual plan as this may influence the results of their athletes in-season.

## Figures and Tables

**Table 1:** Resistance training program.

Training Block	Week	Sets × Reps	Day 1 and 2	Day 3
Strength-Endurance	1	3 × 10	80%	70%
	2	3 × 10	85%	75%
	3	3 × 10	90%	80%
Maximum Strength	4	3 × 5 (1 × 5) *	85%	70%
	5	3 × 5 (1 × 5) *	87.5%	72.5%
	6	3 × 5 (1 × 5) *	92.5%	75%
	7	3 × 5 (1 × 5) *	80%	65%
Overreach	8	5 × 5	85%	75%
Speed-Strength	9	3 × 3 (1 × 5) *	87.5%	67.5%
	10	3 × 2 (1 × 5) *	85%	65%
	11	2 × 2 (1 × 5) *	65% & 60%	---------

* Signifies down set at 50% of target weight after major exercise (squats, bench, mid-thigh pull).

**Table 2:** Resistance training exercise selection.

Training Block	Day 1	Day 2	Day 3
Strength-Endurance	Back Squat, Overhead Press, Bench Press, DB Triceps Ext.	CG MTP, CG SLDL, BB Bent Over Row, DB Bent Lateral Raise	Back Squat, Overhead Press, Bench Press, DB Triceps Ext.
Max Strength	Back Squat, Push Press, Incline Bench Press, Wtd. Dips	CG MTP, Clean Pull, SG SLDL, Pull Ups	Back Squat, Push Press, Incline Bench Press, Wtd. Dips
Overreach	Back Squat, Push Press, DB Step Ups, Bench Press	CG CM Shrug, Clean Pull, CG SLDL, SA DB Bent Over Row	Back Squat, Push Press, DB Step Ups, Bench Press
Speed-Strength	Back Squat + Rocket Jumps, Push Press, Bench press + Med Ball Chest Pass	CG MTP, CG CM Shrug, Vertical Med Ball Toss	Back Squat + Rocket Jumps, Push Press, Bench press + Med Ball Chest Pass

DB = dumbbell, CG = clean grip, MTP = mid-thigh pull, BB = barbell, Ext = extension, Wtd = weighted, SG = snatch grip, SLDL = stiff-legged deadlift, SA = single arm, CM = counter-movement.

**Table 3:** Warm-up protocol to all 1-RM lift attempts and rest time after all warm-up sets.

5 × 30% of 1-RM *	3 × 50% of 1-RM *	2 × 70% of 1-RM *	1 × 80% of 1-RM *	1 × 90% of 1-RM *
1 min	1 min	2 min	3 min	3 min

* 1-RM weight for untrained subjects will be based on the participant’s estimated 1-RM.

**Table 4:** Reliability statistics.

Dependent Variable	%BF	LBM	TBW	LBM_adjusted_	CSA
Intraclass Correlation (ICC)	0.99	0.99	0.99	0.99	0.98
Lower Confidence Limit	0.98	0.98	0.99	0.99	0.96
Upper Confidence Limit	1.00	1.00	1.00	1.00	0.99
Coefficient of Variation (CV) (%)	8.70%	2.67%	2.67%	2.18%	6.33%

**Table 5:** Dependent BIS variables at each time point for high, moderate, low and all subjects.

Group	Variable	Baseline	SE	MS	FOR	Taper
High	BM (kg)	87.7 ± 10.0	89.6 ± 9.4	90.3 ± 9.7	90.5 ± 9.4	88.6 ± 9.2
	%BF	19.4 ± 8.3	19.0 ± 7.9	17.4 ± 8.2	18.5 ± 7.8	17.4 ± 6.8
	TBW (kg)	51.7 ± 4.7	52.9 ± 5.0	54.3 ± 4.6	53.7 ± 5.0	53.3 ± 3.4
	LBM (kg)	70.6 ± 6.5	72.3 ± 6.9	74.2 ± 6.3	73.4 ± 6.8	72.8 ± 4.6
	LBM_adjusted_ (kg)	20.7 ± 1.8	21.1 ± 1.8	21.5 ± 1.7	21.4 ± 1.8	21.1 ± 1.5
Moderate	BM (kg)	100.2 ± 17.9	102.5 ± 20.5	105.4 ± 22.0	105.4 ± 21.5	104.1 ± 22.0
	%BF	25.5 ± 6.4	24.6 ± 7.6	24.9 ± 7.9	25.7 ± 7.5	26.1 ± 7.3
	TBW (kg)	54.4 ± 8.1	56.2 ± 10.8	57.4 ± 10.3	56.8 ± 9.9	55.7 ± 9.4
	LBM (kg)	74.3 ± 11.1	76.8 ± 14.8	78.4 ± 14.1	77.5 ± 13.5	76.1 ± 12.9
	LBM_adjusted_ (kg)	22.5 ± 3.5	23.2 ± 4.3	23.7 ± 4.4	23.6 ± 4.2	23.2 ± 4.2
Low	BM (kg)	83.5 ± 18.5	85.3 ± 18.7	86.3 ± 17.9	86.5 ± 17.9	86.1 ± 18.4
	%BF	22.7 ± 9.7	21.4 ± 10.1	21.7 ± 10.1	21.2 ± 11.0	21.7 ± 9.4
	TBW (kg)	46.1 ± 5.9	48.0 ± 5.5	48.5 ± 5.8	48.7 ± 4.9	48.5 ± 6.1
	LBM (kg)	62.9 ± 8.1	65.5 ± 7.5	66.2 ± 8.0	66.5 ± 6.6	66.3 ± 8.3
	LBM_adjusted_ (kg)	18.9 ± 3.1	19.5 ± 3.1	19.8 ± 3.0	19.8 ± 2.8	19.8 ± 3.2
All Subjects	BM (kg)	89.1 ± 17.0	91.0 ± 17.7 #	92.5 ± 18.2 *#	92.6 ± 18.0 *#	91.5 ± 18.1 #
	%BF	22.6 ± 8.3	21.6 ± 8.6	21.4 ± 8.9	21.7 ± 9.2	21.7 ± 8.4
	TBW (kg)	49.8 ± 6.9	51.5 ± 7.5 #	52.4 ± 7.6 #	52.2 ± 7.0 #	51.7 ± 6.9 #
	LBM (kg)	68.0 ± 9.5	70.3 ± 10.3 #	71.6 ± 10.4 #	71.3 ± 9.6 #	70.6 ± 9.4 #
	LBM_adjusted_ (kg)	20.4 ± 3.1	20.9 ± 3.3 #	21.3 ± 3.4 *#	21.2 ± 3.2 *#	21.0 ± 3.3 #

BM = body mass; %BF= percent body fat; TBW = total body water; LBM = lean body mass; LBM_adjusted_ = lean body mass adjusted for water; * Statistically different from the previous time point (*p* ≤ 0.05). # Statistically different from time point baseline (*p* ≤ 0.05).

**Table 6:** Dependent US variable at each time point for high, moderate, low and all subjects.

Group	Variable	Baseline	SE	MS	FOR	Taper
High	CSA (cm^2^)	33.0 ± 6.3	36.5 ± 6.0	36.3 ± 6.8	36.1 ± 4.6	36.9 ± 3.4
Moderate	CSA (cm^2^)	38.9 ± 12.0	39.8 ± 10.0	42.6 ± 9.8	42.7 ± 9.9	42.3 ± 9.9
Low	CSA (cm^2^)	29.1 ± 6.5	30.8 ± 7.0	32.1 ± 6.8	32.0 ± 5.9	33.1 ± 5.3
All Subjects	CSA (cm^2^)	32.7 ± 8.6	34.7 ± 8.1 #	36.0 ± 8.4 #	36.0 ± 7.8 #	36.3 ± 7.2 #

CSA = Cross-sectional Area. * Statistically different from the previous time point (*p* ≤ 0.05). # Statistically different from time point baseline (*p* ≤ 0.05).

## Data Availability

The data from this study are available upon request from the corresponding author. Data is currently stored in the Sport Science Lab on the campus of East Tennessee State University.

## Supplementary Materials

The following are available online at https://www.mdpi.com/article/10.3390/ijerph18094735/s1, Table S1: Baseline ANOVA of body mass for all subjects; Table S2: Baseline ANOVA of percent body fat for all subjects; Table S3: Baseline ANOVA of total body water for all subjects; Table S4: Baseline ANOVA of lean body mass for all subjects; Table S5: Baseline ANOVA of lean body mass adjusted for all subjects; Table S6: Baseline ANOVA of cross-sectional area for all subjects; Table S7: Post Hoc Comparisons-Status ✻ BM; Table S8: Post Hoc Comparisons-Status ✻ %BF; Table S9: Post Hoc Comparisons-Status ✻ TBW; Table S10: Post Hoc Comparisons-Status ✻ LBM; Table S1: Post Hoc Comparisons-Status ✻ LBMa; Table S12: Post Hoc Comparisons-Status ✻ CSA.
