# Supplementary material for: Lean Body Mass and Muscle Cross-Sectional Area Adaptations Among College Age Males with Different Strength Levels across 11 Weeks of Block Periodized Programmed Resistance Training
Source: Int J Environ Res Public Health. 2021 Apr 29;18(9):4735. doi: 10.3390/ijerph18094735 (PMC8124523; doi:10.3390/ijerph18094735)
Supplement: Supplementary file 1 [file ijerph-18-04735-s001.zip › ijerph-1156879-supplementary.pdf]

Table S.1. Baseline ANOVA of body mass for all subjects

Cases	Sum of Squares	df	Mean Square	F	p
Status	714.912	2	357.456	1.294	0.310
Residuals	3314.112	12	276.176		

Note. Type III Sum of Squares

Table S.2. Baseline ANOVA of percent body fat for all subjects

Cases	Sum of Squares	df	Mean Square	F	p
Status	74.859	2	37.429	0.506	0.615
Residuals	888.422	12	74.035		

Note. Type III Sum of Squares

Table S.3. Baseline ANOVA of total body water for all subjects

Cases	Sum of Squares	df	Mean Square	F	p
Status	234.510	2	117.255	2.559	0.119
Residuals	549.795	12	45.816		

Note. Type III Sum of Squares

Table S.4. Baseline ANOVA of lean body mass for all subjects

Cases	Sum of Squares	df	Mean Square	F	p
Status	437.697	2	218.848	2.559	0.119
Residuals	1026.175	12	85.515		

Note. Type III Sum of Squares

Table S.5. Baseline ANOVA of lean body mass adjusted for all subjects

Cases	Sum of Squares	df	Mean Square	F	p
Status	39.369	2	19.685	2.003	0.178
Residuals	117.906	12	9.825		

Note. Type III Sum of Squares

Table S.6. Baseline ANOVA of cross-sectional area for all subjects

Cases	Sum of Squares	df	Mean Square	F	p
Status	243.940	2	121.970	1.826	0.203
Residuals	801.563	12	66.797		

Note. Type III Sum of Squares

Table S.7. Post Hoc Comparisons - Status ✻ BM

			95% CI for Mean Difference				
		Mean Difference	Lower	Upper	SE	t	p_{holm}
moderate, Baseline	strong, Baseline	12.500	-45.265	70.265	12.218	1.023	1.000
	weak, Baseline	16.632	-34.571	67.835	10.830	1.536	1.000
	moderate, SE	-2.300	-6.062	1.462	1.003	-2.292	1.000
	strong, SE	10.550	-47.215	68.315	12.218	0.863	1.000
	weak, SE	14.918	-36.285	66.121	10.830	1.377	1.000
	moderate, MS	-5.225	-8.987	-1.463	1.003	-5.208	< .001
	strong, MS	9.850	-47.915	67.615	12.218	0.806	1.000
	weak, MS	13.904	-37.299	65.106	10.830	1.284	1.000
	moderate, FOR	-5.225	-8.987	-1.463	1.003	-5.208	< .001
	strong, FOR	9.675	-48.090	67.440	12.218	0.792	1.000
	weak, FOR	13.675	-37.528	64.878	10.830	1.263	1.000
	moderate, Taper	-3.900	-7.662	-0.138	1.003	-3.887	0.032
	strong, Taper	11.600	-46.165	69.365	12.218	0.949	1.000
	weak, Taper	14.104	-37.099	65.306	10.830	1.302	1.000
strong, Baseline	weak, Baseline	4.132	-47.071	55.335	10.830	0.382	1.000
	moderate, SE	-14.800	-72.565	42.965	12.218	-1.211	1.000

Table S.7. Post Hoc Comparisons - Status ✻ BM

		Mean Difference	95% CI for Mean Difference		SE	t	p holm
			Lower	Upper			
	strong, SE	-1.950	-5.712	1.812	1.003	-1.944	1.000
	weak, SE	2.418	-48.785	53.621	10.830	0.223	1.000
	moderate, MS	-17.725	-75.490	40.040	12.218	-1.451	1.000
	strong, MS	-2.650	-6.412	1.112	1.003	-2.641	1.000
	weak, MS	1.404	-49.799	52.606	10.830	0.130	1.000
	moderate, FOR	-17.725	-75.490	40.040	12.218	-1.451	1.000
	strong, FOR	-2.825	-6.587	0.937	1.003	-2.816	0.683
	weak, FOR	1.175	-50.028	52.378	10.830	0.108	1.000
	moderate, Taper	-16.400	-74.165	41.365	12.218	-1.342	1.000
	strong, Taper	-0.900	-4.662	2.862	1.003	-0.897	1.000
	weak, Taper	1.604	-49.599	52.806	10.830	0.148	1.000
weak, Baseline	moderate, SE	-18.932	-70.135	32.271	10.830	-1.748	1.000
	strong, SE	-6.082	-57.285	45.121	10.830	-0.562	1.000
	weak, SE	-1.714	-4.558	1.130	0.758	-2.260	1.000
	moderate, MS	-21.857	-73.060	29.346	10.830	-2.018	1.000
	strong, MS	-6.782	-57.985	44.421	10.830	-0.626	1.000
	weak, MS	-2.729	-5.573	0.115	0.758	-3.598	0.076
	moderate, FOR	-21.857	-73.060	29.346	10.830	-2.018	1.000
	strong, FOR	-6.957	-58.160	44.246	10.830	-0.642	1.000
	weak, FOR	-2.957	-5.801	-0.113	0.758	-3.899	0.031
	moderate, Taper	-20.532	-71.735	30.671	10.830	-1.896	1.000

Table S.7. Post Hoc Comparisons - Status ✻ BM

		Mean Difference	95% CI for Mean Difference		SE	t	p holm
			Lower	Upper			
	strong, Taper	-5.032	-56.235	46.171	10.830	-0.465	1.000
	weak, Taper	-2.529	-5.373	0.315	0.758	-3.334	0.166
moderate, SE	strong, SE	12.850	-44.915	70.615	12.218	1.052	1.000
	weak, SE	17.218	-33.985	68.421	10.830	1.590	1.000
	moderate, MS	-2.925	-6.687	0.837	1.003	-2.915	0.533
	strong, MS	12.150	-45.615	69.915	12.218	0.994	1.000
	weak, MS	16.204	-34.999	67.406	10.830	1.496	1.000
	moderate, FOR	-2.925	-6.687	0.837	1.003	-2.915	0.533
	strong, FOR	11.975	-45.790	69.740	12.218	0.980	1.000
	weak, FOR	15.975	-35.228	67.178	10.830	1.475	1.000
	moderate, Taper	-1.600	-5.362	2.162	1.003	-1.595	1.000
	strong, Taper	13.900	-43.865	71.665	12.218	1.138	1.000
	weak, Taper	16.404	-34.799	67.606	10.830	1.515	1.000
strong, SE	weak, SE	4.368	-46.835	55.571	10.830	0.403	1.000
	moderate, MS	-15.775	-73.540	41.990	12.218	-1.291	1.000
	strong, MS	-0.700	-4.462	3.062	1.003	-0.698	1.000
	weak, MS	3.354	-47.849	54.556	10.830	0.310	1.000
	moderate, FOR	-15.775	-73.540	41.990	12.218	-1.291	1.000
	strong, FOR	-0.875	-4.637	2.887	1.003	-0.872	1.000
	weak, FOR	3.125	-48.078	54.328	10.830	0.289	1.000
	moderate, Taper	-14.450	-72.215	43.315	12.218	-1.183	1.000

Table S.7. Post Hoc Comparisons - Status ✻ BM

		Mean Difference	95% CI for Mean Difference		SE	t	p holm
			Lower	Upper			
	strong, Taper	1.050	-2.712	4.812	1.003	1.047	1.000
	weak, Taper	3.554	-47.649	54.756	10.830	0.328	1.000
weak, SE	moderate, MS	-20.143	-71.346	31.060	10.830	-1.860	1.000
	strong, MS	-5.068	-56.271	46.135	10.830	-0.468	1.000
	weak, MS	-1.014	-3.858	1.830	0.758	-1.337	1.000
	moderate, FOR	-20.143	-71.346	31.060	10.830	-1.860	1.000
	strong, FOR	-5.243	-56.446	45.960	10.830	-0.484	1.000
	weak, FOR	-1.243	-4.087	1.601	0.758	-1.639	1.000
	moderate, Taper	-18.818	-70.021	32.385	10.830	-1.738	1.000
	strong, Taper	-3.318	-54.521	47.885	10.830	-0.306	1.000
	weak, Taper	-0.814	-3.658	2.030	0.758	-1.074	1.000
moderate, MS	strong, MS	15.075	-42.690	72.840	12.218	1.234	1.000
	weak, MS	19.129	-32.074	70.331	10.830	1.766	1.000
	moderate, FOR	5.884e -15	-3.762	3.762	1.003	5.865e -15	1.000
	strong, FOR	14.900	-42.865	72.665	12.218	1.220	1.000
	weak, FOR	18.900	-32.303	70.103	10.830	1.745	1.000
	moderate, Taper	1.325	-2.437	5.087	1.003	1.321	1.000
	strong, Taper	16.825	-40.940	74.590	12.218	1.377	1.000
	weak, Taper	19.329	-31.874	70.531	10.830	1.785	1.000
strong, MS	weak, MS	4.054	-47.149	55.256	10.830	0.374	1.000
	moderate, FOR	-15.075	-72.840	42.690	12.218	-1.234	1.000

Table S.7. Post Hoc Comparisons - Status ✻ BM

		Mean Difference	95% CI for Mean Difference		SE	t	p holm
			Lower	Upper			
	strong, FOR	-0.175	-3.937	3.587	1.003	-0.174	1.000
	weak, FOR	3.825	-47.378	55.028	10.830	0.353	1.000
	moderate, Taper	-13.750	-71.515	44.015	12.218	-1.125	1.000
	strong, Taper	1.750	-2.012	5.512	1.003	1.744	1.000
	weak, Taper	4.254	-46.949	55.456	10.830	0.393	1.000
weak, MS	moderate, FOR	-19.129	-70.331	32.074	10.830	-1.766	1.000
	strong, FOR	-4.229	-55.431	46.974	10.830	-0.390	1.000
	weak, FOR	-0.229	-3.073	2.615	0.758	-0.301	1.000
	moderate, Taper	-17.804	-69.006	33.399	10.830	-1.644	1.000
	strong, Taper	-2.304	-53.506	48.899	10.830	-0.213	1.000
	weak, Taper	0.200	-2.644	3.044	0.758	0.264	1.000
moderate, FOR	strong, FOR	14.900	-42.865	72.665	12.218	1.220	1.000
	weak, FOR	18.900	-32.303	70.103	10.830	1.745	1.000
	moderate, Taper	1.325	-2.437	5.087	1.003	1.321	1.000
	strong, Taper	16.825	-40.940	74.590	12.218	1.377	1.000
	weak, Taper	19.329	-31.874	70.531	10.830	1.785	1.000
strong, FOR	weak, FOR	4.000	-47.203	55.203	10.830	0.369	1.000
	moderate, Taper	-13.575	-71.340	44.190	12.218	-1.111	1.000
	strong, Taper	1.925	-1.837	5.687	1.003	1.919	1.000
	weak, Taper	4.429	-46.774	55.631	10.830	0.409	1.000

Table S.7. Post Hoc Comparisons - Status ✻ BM

		Mean Difference	95% CI for Mean Difference		SE	t	p holm
			Lower	Upper			
weak, FOR	moderate, Taper	-17.575	-68.778	33.628	10.830	-1.623	1.000
	strong, Taper	-2.075	-53.278	49.128	10.830	-0.192	1.000
	weak, Taper	0.429	-2.415	3.273	0.758	0.565	1.000
moderate, Taper	strong, Taper	15.500	-42.265	73.265	12.218	1.269	1.000
	weak, Taper	18.004	-33.199	69.206	10.830	1.662	1.000
strong, Taper	weak, Taper	2.504	-48.699	53.706	10.830	0.231	1.000

Note. P-value and confidence intervals adjusted for comparing a family of 105 estimates (confidence intervals corrected using the bonferroni method).

Table S.8. Post Hoc Comparisons - Status ✻ %BF

		Mean Difference	95% CI for Mean Difference		SE	t	p holm
			Lower	Upper			
moderate, Baseline	strong, Baseline	6.110	-23.264	35.484	6.294	0.971	1.000
	weak, Baseline	2.827	-23.210	28.864	5.579	0.507	1.000
	moderate, SE	0.918	-3.263	5.098	1.115	0.823	1.000
	strong, SE	6.545	-22.829	35.919	6.294	1.040	1.000
	weak, SE	4.144	-21.893	30.181	5.579	0.743	1.000
	moderate, MS	0.593	-3.588	4.773	1.115	0.531	1.000
	strong, MS	8.123	-21.251	37.496	6.294	1.290	1.000
	weak, MS	3.854	-22.183	29.891	5.579	0.691	1.000
	moderate, FOR	-0.212	-4.393	3.968	1.115	-0.191	1.000

Table S.8. Post Hoc Comparisons - Status ✻ %BF

		Mean Difference	95% CI for Mean Difference		SE	t	p_{holm}
			Lower	Upper			
	strong, FOR	7.015	-22.359	36.389	6.294	1.115	1.000
	weak, FOR	4.301	-21.736	30.338	5.579	0.771	1.000
	moderate, Taper	-0.617	-4.798	3.563	1.115	-0.554	1.000
	strong, Taper	8.113	-21.261	37.486	6.294	1.289	1.000
	weak, Taper	3.865	-22.172	29.903	5.579	0.693	1.000
strong, Baseline	weak, Baseline	-3.283	-29.320	22.754	5.579	-0.588	1.000
	moderate, SE	-5.192	-34.566	24.181	6.294	-0.825	1.000
	strong, SE	0.435	-3.746	4.616	1.115	0.390	1.000
	weak, SE	-1.966	-28.003	24.071	5.579	-0.352	1.000
	moderate, MS	-5.517	-34.891	23.856	6.294	-0.877	1.000
	strong, MS	2.013	-2.168	6.193	1.115	1.805	1.000
	weak, MS	-2.256	-28.293	23.781	5.579	-0.404	1.000
	moderate, FOR	-6.322	-35.696	23.051	6.294	-1.005	1.000
	strong, FOR	0.905	-3.276	5.086	1.115	0.812	1.000
	weak, FOR	-1.809	-27.846	24.228	5.579	-0.324	1.000
	moderate, Taper	-6.727	-36.101	22.646	6.294	-1.069	1.000
	strong, Taper	2.003	-2.178	6.183	1.115	1.796	1.000
	weak, Taper	-2.245	-28.282	23.793	5.579	-0.402	1.000
weak, Baseline	moderate, SE	-1.909	-27.947	24.128	5.579	-0.342	1.000
	strong, SE	3.718	-22.319	29.755	5.579	0.666	1.000
	weak, SE	1.317	-1.843	4.478	0.843	1.563	1.000
	moderate, MS	-2.234	-28.272	23.803	5.579	-0.400	1.000
	strong, MS	5.296	-20.742	31.333	5.579	0.949	1.000

Table S.8. Post Hoc Comparisons - Status ✻ %BF

		Mean Difference	95% CI for Mean Difference		SE	t	p holm
			Lower	Upper			
	weak, MS	1.027	-2.133	4.188	0.843	1.219	1.000
	moderate, FOR	-3.039	-29.077	22.998	5.579	-0.545	1.000
	strong, FOR	4.188	-21.849	30.225	5.579	0.751	1.000
	weak, FOR	1.474	-1.686	4.635	0.843	1.749	1.000
	moderate, Taper	-3.444	-29.482	22.593	5.579	-0.617	1.000
	strong, Taper	5.286	-20.752	31.323	5.579	0.947	1.000
	weak, Taper	1.039	-2.122	4.199	0.843	1.232	1.000
moderate, SE	strong, SE	5.627	-23.746	35.001	6.294	0.894	1.000
	weak, SE	3.226	-22.811	29.264	5.579	0.578	1.000
	moderate, MS	-0.325	-4.506	3.856	1.115	-0.291	1.000
	strong, MS	7.205	-22.169	36.579	6.294	1.145	1.000
	weak, MS	2.936	-23.101	28.974	5.579	0.526	1.000
	moderate, FOR	-1.130	-5.311	3.051	1.115	-1.013	1.000
	strong, FOR	6.097	-23.276	35.471	6.294	0.969	1.000
	weak, FOR	3.384	-22.654	29.421	5.579	0.606	1.000
	moderate, Taper	-1.535	-5.716	2.646	1.115	-1.377	1.000
	strong, Taper	7.195	-22.179	36.569	6.294	1.143	1.000
	weak, Taper	2.948	-23.089	28.985	5.579	0.528	1.000
strong, SE	weak, SE	-2.401	-28.438	23.636	5.579	-0.430	1.000
	moderate, MS	-5.952	-35.326	23.421	6.294	-0.946	1.000
	strong, MS	1.578	-2.603	5.758	1.115	1.415	1.000
	weak, MS	-2.691	-28.728	23.346	5.579	-0.482	1.000
	moderate, FOR	-6.757	-36.131	22.616	6.294	-1.074	1.000

Table S.8. Post Hoc Comparisons - Status ✻ %BF

			95% CI for Mean Difference				
		**Mean Difference**	**Lower**	**Upper**	**SE**	**t**	**p holm**
	strong, FOR	0.470	-3.711	4.651	1.115	0.422	1.000
	weak, FOR	-2.244	-28.281	23.793	5.579	-0.402	1.000
	moderate, Taper	-7.162	-36.536	22.211	6.294	-1.138	1.000
	strong, Taper	1.568	-2.613	5.748	1.115	1.406	1.000
	weak, Taper	-2.680	-28.717	23.358	5.579	-0.480	1.000
weak, SE	moderate, MS	-3.551	-29.589	22.486	5.579	-0.637	1.000
	strong, MS	3.979	-22.059	30.016	5.579	0.713	1.000
	weak, MS	-0.290	-3.450	2.870	0.843	-0.344	1.000
	moderate, FOR	-4.356	-30.394	21.681	5.579	-0.781	1.000
	strong, FOR	2.871	-23.166	28.908	5.579	0.515	1.000
	weak, FOR	0.157	-3.003	3.318	0.843	0.186	1.000
	moderate, Taper	-4.761	-30.799	21.276	5.579	-0.853	1.000
	strong, Taper	3.969	-22.069	30.006	5.579	0.711	1.000
	weak, Taper	-0.279	-3.439	2.882	0.843	-0.331	1.000
moderate, MS	strong, MS	7.530	-21.844	36.904	6.294	1.196	1.000
	weak, MS	3.261	-22.776	29.299	5.579	0.585	1.000
	moderate, FOR	-0.805	-4.986	3.376	1.115	-0.722	1.000
	strong, FOR	6.422	-22.951	35.796	6.294	1.020	1.000
	weak, FOR	3.709	-22.329	29.746	5.579	0.665	1.000
	moderate, Taper	-1.210	-5.391	2.971	1.115	-1.085	1.000
	strong, Taper	7.520	-21.854	36.894	6.294	1.195	1.000
	weak, Taper	3.273	-22.764	29.310	5.579	0.587	1.000
strong, MS	weak, MS	-4.269	-30.306	21.769	5.579	-0.765	1.000

Table S.8. Post Hoc Comparisons - Status ✻ %BF

		Mean Difference	95% CI for Mean Difference		SE	t	p holm
			Lower	Upper			
	moderate, FOR	-8.335	-37.709	21.039	6.294	-1.324	1.000
	strong, FOR	-1.108	-5.288	3.073	1.115	-0.993	1.000
	weak, FOR	-3.821	-29.859	22.216	5.579	-0.685	1.000
	moderate, Taper	-8.740	-38.114	20.634	6.294	-1.389	1.000
	strong, Taper	-0.010	-4.191	4.171	1.115	-0.009	1.000
	weak, Taper	-4.257	-30.294	21.780	5.579	-0.763	1.000
weak, MS	moderate, FOR	-4.066	-30.104	21.971	5.579	-0.729	1.000
	strong, FOR	3.161	-22.876	29.198	5.579	0.567	1.000
	weak, FOR	0.447	-2.713	3.608	0.843	0.531	1.000
	moderate, Taper	-4.471	-30.509	21.566	5.579	-0.801	1.000
	strong, Taper	4.259	-21.779	30.296	5.579	0.763	1.000
	weak, Taper	0.011	-3.149	3.172	0.843	0.014	1.000
moderate, FOR	strong, FOR	7.227	-22.146	36.601	6.294	1.148	1.000
	weak, FOR	4.514	-21.524	30.551	5.579	0.809	1.000
	moderate, Taper	-0.405	-4.586	3.776	1.115	-0.363	1.000
	strong, Taper	8.325	-21.049	37.699	6.294	1.323	1.000
	weak, Taper	4.078	-21.959	30.115	5.579	0.731	1.000
strong, FOR	weak, FOR	-2.714	-28.751	23.323	5.579	-0.486	1.000
	moderate, Taper	-7.632	-37.006	21.741	6.294	-1.213	1.000
	strong, Taper	1.098	-3.083	5.278	1.115	0.984	1.000
	weak, Taper	-3.150	-29.187	22.888	5.579	-0.565	1.000
weak, FOR	moderate, Taper	-4.919	-30.956	21.119	5.579	-0.882	1.000
	strong, Taper	3.811	-22.226	29.849	5.579	0.683	1.000

Table S.8. Post Hoc Comparisons - Status ✻ %BF

			95% CI for Mean Difference				
		Mean Difference	Lower	Upper	SE	t	p_{holm}
	weak, Taper	-0.436	-3.596	2.725	0.843	-0.517	1.000
moderate, Taper	strong, Taper	8.730	-20.644	38.104	6.294	1.387	1.000
	weak, Taper	4.483	-21.554	30.520	5.579	0.804	1.000
strong, Taper	weak, Taper	-4.247	-30.284	21.790	5.579	-0.761	1.000

Note. P-value and confidence intervals adjusted for comparing a family of 105 estimates (confidence intervals corrected using the bonferroni method).

Table S.9. Post Hoc Comparisons - Status ✻ TBW

			95% CI for Mean Difference				
		Mean Difference	Lower	Upper	SE	t	p_{holm}
moderate, Baseline	strong, Baseline	2.718	-19.667	25.102	4.810	0.565	1.000
	weak, Baseline	9.031	-10.811	28.873	4.263	2.118	1.000
	moderate, SE	-1.833	-5.284	1.619	0.920	-1.991	1.000
	strong, SE	1.460	-20.925	23.845	4.810	0.304	1.000
	weak, SE	6.399	-13.443	26.241	4.263	1.501	1.000
	moderate, MS	-2.985	-6.436	0.466	0.920	-3.243	0.218
	strong, MS	0.030	-22.355	22.415	4.810	0.006	1.000
	weak, MS	5.901	-13.941	25.743	4.263	1.384	1.000
	moderate, FOR	-2.393	-5.844	1.059	0.920	-2.599	1.000
	strong, FOR	0.632	-21.752	23.017	4.810	0.132	1.000
	weak, FOR	5.706	-14.136	25.549	4.263	1.339	1.000
	moderate, Taper	-1.302	-4.754	2.149	0.920	-1.415	1.000

Table S.9. Post Hoc Comparisons - Status ✻ TBW

			95% CI for Mean Difference				
		Mean Difference	Lower	Upper	SE	t	p_{holm}
	strong, Taper	1.095	-21.290	23.480	4.810	0.228	1.000
	weak, Taper	5.821	-14.021	25.663	4.263	1.365	1.000
strong, Baseline	weak, Baseline	6.313	-13.529	26.155	4.263	1.481	1.000
	moderate, SE	-4.550	-26.935	17.835	4.810	-0.946	1.000
	strong, SE	-1.258	-4.709	2.194	0.920	-1.366	1.000
	weak, SE	3.682	-16.160	23.524	4.263	0.864	1.000
	moderate, MS	-5.703	-28.087	16.682	4.810	-1.186	1.000
	strong, MS	-2.688	-6.139	0.764	0.920	-2.920	0.532
	weak, MS	3.183	-16.659	23.025	4.263	0.747	1.000
	moderate, FOR	-5.110	-27.495	17.275	4.810	-1.062	1.000
	strong, FOR	-2.085	-5.536	1.366	0.920	-2.265	1.000
	weak, FOR	2.989	-16.853	22.831	4.263	0.701	1.000
	moderate, Taper	-4.020	-26.405	18.365	4.810	-0.836	1.000
	strong, Taper	-1.623	-5.074	1.829	0.920	-1.763	1.000
	weak, Taper	3.103	-16.739	22.945	4.263	0.728	1.000
weak, Baseline	moderate, SE	-10.863	-30.705	8.979	4.263	-2.548	1.000
	strong, SE	-7.571	-27.413	12.271	4.263	-1.776	1.000
	weak, SE	-2.631	-5.240	-0.022	0.696	-3.782	0.044
	moderate, MS	-12.016	-31.858	7.826	4.263	-2.818	1.000
	strong, MS	-9.001	-28.843	10.841	4.263	-2.111	1.000
	weak, MS	-3.130	-5.739	-0.521	0.696	-4.499	0.004
	moderate, FOR	-11.423	-31.265	8.419	4.263	-2.679	1.000
	strong, FOR	-8.398	-28.240	11.444	4.263	-1.970	1.000

Table S.9. Post Hoc Comparisons - Status ✻ TBW

			95% CI for Mean Difference				
		Mean Difference	Lower	Upper	SE	t	p holm
	weak, FOR	-3.324	-5.933	-0.715	0.696	-4.778	0.002
	moderate, Taper	-10.333	-30.175	9.509	4.263	-2.424	1.000
	strong, Taper	-7.936	-27.778	11.906	4.263	-1.861	1.000
	weak, Taper	-3.210	-5.819	-0.601	0.696	-4.614	0.003
moderate, SE	strong, SE	3.292	-19.092	25.677	4.810	0.685	1.000
	weak, SE	8.232	-11.610	28.074	4.263	1.931	1.000
	moderate, MS	-1.153	-4.604	2.299	0.920	-1.252	1.000
	strong, MS	1.862	-20.522	24.247	4.810	0.387	1.000
	weak, MS	7.733	-12.109	27.575	4.263	1.814	1.000
	moderate, FOR	-0.560	-4.011	2.891	0.920	-0.608	1.000
	strong, FOR	2.465	-19.920	24.850	4.810	0.513	1.000
	weak, FOR	7.539	-12.303	27.381	4.263	1.768	1.000
	moderate, Taper	0.530	-2.921	3.981	0.920	0.576	1.000
	strong, Taper	2.927	-19.457	25.312	4.810	0.609	1.000
	weak, Taper	7.653	-12.189	27.495	4.263	1.795	1.000
strong, SE	weak, SE	4.939	-14.903	24.781	4.263	1.159	1.000
	moderate, MS	-4.445	-26.830	17.940	4.810	-0.924	1.000
	strong, MS	-1.430	-4.881	2.021	0.920	-1.554	1.000
	weak, MS	4.441	-15.401	24.283	4.263	1.042	1.000
	moderate, FOR	-3.852	-26.237	18.532	4.810	-0.801	1.000
	strong, FOR	-0.827	-4.279	2.624	0.920	-0.899	1.000
	weak, FOR	4.246	-15.596	24.089	4.263	0.996	1.000
	moderate, Taper	-2.762	-25.147	19.622	4.810	-0.574	1.000

Table S.9. Post Hoc Comparisons - Status ✻ TBW

		Mean Difference	95% CI for Mean Difference		SE	t	p_{holm}
			Lower	Upper			
	strong, Taper	-0.365	-3.816	3.086	0.920	-0.397	1.000
	weak, Taper	4.361	-15.481	24.203	4.263	1.023	1.000
weak, SE	moderate, MS	-9.384	-29.226	10.458	4.263	-2.201	1.000
	strong, MS	-6.369	-26.211	13.473	4.263	-1.494	1.000
	weak, MS	-0.499	-3.108	2.110	0.696	-0.717	1.000
	moderate, FOR	-8.792	-28.634	11.050	4.263	-2.062	1.000
	strong, FOR	-5.767	-25.609	14.075	4.263	-1.353	1.000
	weak, FOR	-0.693	-3.302	1.916	0.696	-0.996	1.000
	moderate, Taper	-7.702	-27.544	12.140	4.263	-1.807	1.000
	strong, Taper	-5.304	-25.146	14.538	4.263	-1.244	1.000
	weak, Taper	-0.579	-3.188	2.030	0.696	-0.832	1.000
moderate, MS	strong, MS	3.015	-19.370	25.400	4.810	0.627	1.000
	weak, MS	8.886	-10.956	28.728	4.263	2.084	1.000
	moderate, FOR	0.593	-2.859	4.044	0.920	0.644	1.000
	strong, FOR	3.617	-18.767	26.002	4.810	0.752	1.000
	weak, FOR	8.691	-11.151	28.534	4.263	2.039	1.000
	moderate, Taper	1.683	-1.769	5.134	0.920	1.828	1.000
	strong, Taper	4.080	-18.305	26.465	4.810	0.848	1.000
	weak, Taper	8.806	-11.036	28.648	4.263	2.066	1.000
strong, MS	weak, MS	5.871	-13.971	25.713	4.263	1.377	1.000
	moderate, FOR	-2.422	-24.807	19.962	4.810	-0.504	1.000
	strong, FOR	0.603	-2.849	4.054	0.920	0.655	1.000
	weak, FOR	5.676	-14.166	25.519	4.263	1.331	1.000

Table S.9. Post Hoc Comparisons - Status ✻ TBW

			95% CI for Mean Difference				
		Mean Difference	Lower	Upper	SE	t	p holm
	moderate, Taper	-1.332	-23.717	21.052	4.810	-0.277	1.000
	strong, Taper	1.065	-2.386	4.516	0.920	1.157	1.000
	weak, Taper	5.791	-14.051	25.633	4.263	1.358	1.000
weak, MS	moderate, FOR	-8.293	-28.135	11.549	4.263	-1.945	1.000
	strong, FOR	-5.268	-25.110	14.574	4.263	-1.236	1.000
	weak, FOR	-0.194	-2.803	2.415	0.696	-0.279	1.000
	moderate, Taper	-7.203	-27.045	12.639	4.263	-1.690	1.000
	strong, Taper	-4.806	-24.648	15.036	4.263	-1.127	1.000
	weak, Taper	-0.080	-2.689	2.529	0.696	-0.115	1.000
moderate, FOR	strong, FOR	3.025	-19.360	25.410	4.810	0.629	1.000
	weak, FOR	8.099	-11.743	27.941	4.263	1.900	1.000
	moderate, Taper	1.090	-2.361	4.541	0.920	1.184	1.000
	strong, Taper	3.487	-18.897	25.872	4.810	0.725	1.000
	weak, Taper	8.213	-11.629	28.055	4.263	1.927	1.000
strong, FOR	weak, FOR	5.074	-14.768	24.916	4.263	1.190	1.000
	moderate, Taper	-1.935	-24.320	20.450	4.810	-0.402	1.000
	strong, Taper	0.463	-2.989	3.914	0.920	0.502	1.000
	weak, Taper	5.188	-14.654	25.030	4.263	1.217	1.000
weak, FOR	moderate, Taper	-7.009	-26.851	12.833	4.263	-1.644	1.000
	strong, Taper	-4.611	-24.454	15.231	4.263	-1.082	1.000
	weak, Taper	0.114	-2.495	2.723	0.696	0.164	1.000
moderate, Taper	strong, Taper	2.397	-19.987	24.782	4.810	0.498	1.000
	weak, Taper	7.123	-12.719	26.965	4.263	1.671	1.000

Table S.9. Post Hoc Comparisons - Status ✻ TBW

			95% CI for Mean Difference				
		Mean Difference	Lower	Upper	SE	t	p holm
strong, Taper	weak, Taper	4.726	-15.116	24.568	4.263	1.108	1.000

Note. P-value and confidence intervals adjusted for comparing a family of 105 estimates (confidence intervals corrected using the bonferroni method).

Table S.10. Post Hoc Comparisons - Status ✻ LBM

			95% CI for Mean Difference				
		Mean Difference	Lower	Upper	SE	t	p holm
moderate, Baseline	strong, Baseline	3.713	-26.874	34.299	6.572	0.565	1.000
	weak, Baseline	12.337	-14.774	39.449	5.825	2.118	1.000
	moderate, SE	-2.503	-7.220	2.215	1.258	-1.989	1.000
	strong, SE	1.990	-28.596	32.576	6.572	0.303	1.000
	weak, SE	8.742	-18.370	35.854	5.825	1.501	1.000
	moderate, MS	-4.080	-8.797	0.637	1.258	-3.243	0.218
	strong, MS	0.037	-30.549	30.624	6.572	0.006	1.000
	weak, MS	8.062	-19.050	35.174	5.825	1.384	1.000
	moderate, FOR	-3.275	-7.992	1.442	1.258	-2.603	1.000
	strong, FOR	0.850	-29.736	31.436	6.572	0.129	1.000
	weak, FOR	7.792	-19.320	34.904	5.825	1.338	1.000
	moderate, Taper	-1.778	-6.495	2.940	1.258	-1.413	1.000
	strong, Taper	1.497	-29.089	32.084	6.572	0.228	1.000
	weak, Taper	7.950	-19.162	35.062	5.825	1.365	1.000
strong, Baseline	weak, Baseline	8.625	-18.487	35.737	5.825	1.481	1.000

Table S.10. Post Hoc Comparisons - Status ✻ LBM

		Mean Difference	95% CI for Mean Difference Lower	Upper	SE	t	p holm
	moderate, SE	-6.215	-36.801	24.371	6.572	-0.946	1.000
	strong, SE	-1.723	-6.440	2.995	1.258	-1.369	1.000
	weak, SE	5.029	-22.083	32.141	5.825	0.863	1.000
	moderate, MS	-7.793	-38.379	22.794	6.572	-1.186	1.000
	strong, MS	-3.675	-8.392	1.042	1.258	-2.921	0.530
	weak, MS	4.349	-22.763	31.461	5.825	0.747	1.000
	moderate, FOR	-6.988	-37.574	23.599	6.572	-1.063	1.000
	strong, FOR	-2.863	-7.580	1.855	1.258	-2.275	1.000
	weak, FOR	4.079	-23.033	31.191	5.825	0.700	1.000
	moderate, Taper	-5.490	-36.076	25.096	6.572	-0.835	1.000
	strong, Taper	-2.215	-6.932	2.502	1.258	-1.761	1.000
	weak, Taper	4.238	-22.874	31.350	5.825	0.728	1.000
weak, Baseline	moderate, SE	-14.840	-41.952	12.272	5.825	-2.548	1.000
	strong, SE	-10.348	-37.459	16.764	5.825	-1.776	1.000
	weak, SE	-3.596	-7.162	-0.030	0.951	-3.781	0.044
	moderate, MS	-16.418	-43.529	10.694	5.825	-2.818	1.000
	strong, MS	-12.300	-39.412	14.812	5.825	-2.112	1.000
	weak, MS	-4.276	-7.842	-0.710	0.951	-4.496	0.005
	moderate, FOR	-15.612	-42.724	11.499	5.825	-2.680	1.000
	strong, FOR	-11.488	-38.599	15.624	5.825	-1.972	1.000
	weak, FOR	-4.546	-8.112	-0.980	0.951	-4.780	0.002
	moderate, Taper	-14.115	-41.227	12.997	5.825	-2.423	1.000
	strong, Taper	-10.840	-37.952	16.272	5.825	-1.861	1.000

Table S.10. Post Hoc Comparisons - Status ✻ LBM

		Mean Difference	95% CI for Mean Difference		SE	t	p holm
			Lower	Upper			
	weak, Taper	-4.387	-7.953	-0.821	0.951	-4.613	0.003
moderate, SE	strong, SE	4.492	-26.094	35.079	6.572	0.684	1.000
	weak, SE	11.244	-15.868	38.356	5.825	1.930	1.000
	moderate, MS	-1.578	-6.295	3.140	1.258	-1.254	1.000
	strong, MS	2.540	-28.046	33.126	6.572	0.387	1.000
	weak, MS	10.564	-16.548	37.676	5.825	1.814	1.000
	moderate, FOR	-0.772	-5.490	3.945	1.258	-0.614	1.000
	strong, FOR	3.352	-27.234	33.939	6.572	0.510	1.000
	weak, FOR	10.294	-16.818	37.406	5.825	1.767	1.000
	moderate, Taper	0.725	-3.992	5.442	1.258	0.576	1.000
	strong, Taper	4.000	-26.586	34.586	6.572	0.609	1.000
	weak, Taper	10.453	-16.659	37.565	5.825	1.794	1.000
strong, SE	weak, SE	6.752	-20.360	33.864	5.825	1.159	1.000
	moderate, MS	-6.070	-36.656	24.516	6.572	-0.924	1.000
	strong, MS	-1.952	-6.670	2.765	1.258	-1.552	1.000
	weak, MS	6.072	-21.040	33.184	5.825	1.042	1.000
	moderate, FOR	-5.265	-35.851	25.321	6.572	-0.801	1.000
	strong, FOR	-1.140	-5.857	3.577	1.258	-0.906	1.000
	weak, FOR	5.802	-21.310	32.914	5.825	0.996	1.000
	moderate, Taper	-3.767	-34.354	26.819	6.572	-0.573	1.000
	strong, Taper	-0.492	-5.210	4.225	1.258	-0.391	1.000
	weak, Taper	5.960	-21.152	33.072	5.825	1.023	1.000
weak, SE	moderate, MS	-12.822	-39.934	14.290	5.825	-2.201	1.000

Table S.10. Post Hoc Comparisons - Status ✻ LBM

			95% CI for Mean Difference				
		Mean Difference	Lower	Upper	SE	t	p_{holm}
	strong, MS	-8.704	-35.816	18.408	5.825	-1.494	1.000
	weak, MS	-0.680	-4.246	2.886	0.951	-0.715	1.000
	moderate, FOR	-12.017	-39.129	15.095	5.825	-2.063	1.000
	strong, FOR	-7.892	-35.004	19.220	5.825	-1.355	1.000
	weak, FOR	-0.950	-4.516	2.616	0.951	-0.999	1.000
	moderate, Taper	-10.519	-37.631	16.593	5.825	-1.806	1.000
	strong, Taper	-7.244	-34.356	19.868	5.825	-1.244	1.000
	weak, Taper	-0.791	-4.357	2.775	0.951	-0.832	1.000
moderate, MS	strong, MS	4.117	-26.469	34.704	6.572	0.627	1.000
	weak, MS	12.142	-14.970	39.254	5.825	2.084	1.000
	moderate, FOR	0.805	-3.912	5.522	1.258	0.640	1.000
	strong, FOR	4.930	-25.656	35.516	6.572	0.750	1.000
	weak, FOR	11.872	-15.240	38.984	5.825	2.038	1.000
	moderate, Taper	2.303	-2.415	7.020	1.258	1.830	1.000
	strong, Taper	5.577	-25.009	36.164	6.572	0.849	1.000
	weak, Taper	12.030	-15.082	39.142	5.825	2.065	1.000
strong, MS	weak, MS	8.024	-19.088	35.136	5.825	1.378	1.000
	moderate, FOR	-3.312	-33.899	27.274	6.572	-0.504	1.000
	strong, FOR	0.813	-3.905	5.530	1.258	0.646	1.000
	weak, FOR	7.754	-19.358	34.866	5.825	1.331	1.000
	moderate, Taper	-1.815	-32.401	28.771	6.572	-0.276	1.000
	strong, Taper	1.460	-3.257	6.177	1.258	1.161	1.000
	weak, Taper	7.913	-19.199	35.025	5.825	1.358	1.000

Table S.10. Post Hoc Comparisons - Status ✻ LBM

		Mean Difference	95% CI for Mean Difference		SE	t	p holm
			Lower	Upper			
weak, MS	moderate, FOR	-11.337	-38.449	15.775	5.825	-1.946	1.000
	strong, FOR	-7.212	-34.324	19.900	5.825	-1.238	1.000
	weak, FOR	-0.270	-3.836	3.296	0.951	-0.284	1.000
	moderate, Taper	-9.839	-36.951	17.273	5.825	-1.689	1.000
	strong, Taper	-6.564	-33.676	20.548	5.825	-1.127	1.000
	weak, Taper	-0.111	-3.677	3.455	0.951	-0.117	1.000
moderate, FOR	strong, FOR	4.125	-26.461	34.711	6.572	0.628	1.000
	weak, FOR	11.067	-16.045	38.179	5.825	1.900	1.000
	moderate, Taper	1.498	-3.220	6.215	1.258	1.190	1.000
	strong, Taper	4.772	-25.814	35.359	6.572	0.726	1.000
	weak, Taper	11.225	-15.887	38.337	5.825	1.927	1.000
strong, FOR	weak, FOR	6.942	-20.170	34.054	5.825	1.192	1.000
	moderate, Taper	-2.627	-33.214	27.959	6.572	-0.400	1.000
	strong, Taper	0.647	-4.070	5.365	1.258	0.515	1.000
	weak, Taper	7.100	-20.012	34.212	5.825	1.219	1.000
weak, FOR	moderate, Taper	-9.569	-36.681	17.543	5.825	-1.643	1.000
	strong, Taper	-6.294	-33.406	20.818	5.825	-1.081	1.000
	weak, Taper	0.159	-3.407	3.725	0.951	0.167	1.000
moderate, Taper	strong, Taper	3.275	-27.311	33.861	6.572	0.498	1.000
	weak, Taper	9.728	-17.384	36.840	5.825	1.670	1.000
strong, Taper	weak, Taper	6.453	-20.659	33.565	5.825	1.108	1.000

Note. P-value and confidence intervals adjusted for comparing a family of 105 estimates (confidence intervals corrected using the bonferroni method).

Table S.11. Post Hoc Comparisons - Status ✻ LBMa

		Mean Difference	95% CI for Mean Difference		SE	t	p_{holm}
			Lower	Upper			
moderate, Baseline	strong, Baseline	1.860	-8.584	12.304	2.223	0.837	1.000
	weak, Baseline	3.878	-5.380	13.136	1.971	1.968	1.000
	moderate, SE	-0.633	-1.770	0.505	0.303	-2.085	1.000
	strong, SE	1.405	-9.039	11.849	2.223	0.632	1.000
	weak, SE	2.978	-6.280	12.236	1.971	1.511	1.000
	moderate, MS	-1.190	-2.328	-0.052	0.303	-3.922	0.028
	strong, MS	1.005	-9.439	11.449	2.223	0.452	1.000
	weak, MS	2.762	-6.495	12.020	1.971	1.402	1.000
	moderate, FOR	-1.055	-2.193	0.083	0.303	-3.477	0.109
	strong, FOR	1.122	-9.322	11.567	2.223	0.505	1.000
	weak, FOR	2.707	-6.551	11.965	1.971	1.374	1.000
	moderate, Taper	-0.670	-1.808	0.468	0.303	-2.208	1.000
	strong, Taper	1.430	-9.014	11.874	2.223	0.643	1.000
	weak, Taper	2.741	-6.517	11.999	1.971	1.391	1.000
strong, Baseline	weak, Baseline	2.018	-7.240	11.276	1.971	1.024	1.000
	moderate, SE	-2.492	-12.937	7.952	2.223	-1.121	1.000
	strong, SE	-0.455	-1.593	0.683	0.303	-1.500	1.000
	weak, SE	1.118	-8.140	10.376	1.971	0.567	1.000
	moderate, MS	-3.050	-13.494	7.394	2.223	-1.372	1.000
	strong, MS	-0.855	-1.993	0.283	0.303	-2.818	0.693
	weak, MS	0.903	-8.355	10.160	1.971	0.458	1.000
	moderate, FOR	-2.915	-13.359	7.529	2.223	-1.311	1.000

Table S.11. Post Hoc Comparisons - Status ✻ LBMa

		Mean Difference	95% CI for Mean Difference		SE	t	p holm
			Lower	Upper			
	strong, FOR	-0.737	-1.875	0.400	0.303	-2.431	1.000
	weak, FOR	0.847	-8.411	10.105	1.971	0.430	1.000
	moderate, Taper	-2.530	-12.974	7.914	2.223	-1.138	1.000
	strong, Taper	-0.430	-1.568	0.708	0.303	-1.417	1.000
	weak, Taper	0.881	-8.377	10.139	1.971	0.447	1.000
weak, Baseline	moderate, SE	-4.511	-13.769	4.747	1.971	-2.289	1.000
	strong, SE	-2.473	-11.731	6.785	1.971	-1.255	1.000
	weak, SE	-0.900	-1.760	-0.040	0.229	-3.924	0.028
	moderate, MS	-5.068	-14.326	4.190	1.971	-2.572	1.000
	strong, MS	-2.873	-12.131	6.385	1.971	-1.458	1.000
	weak, MS	-1.116	-1.976	-0.256	0.229	-4.865	0.001
	moderate, FOR	-4.933	-14.191	4.325	1.971	-2.503	1.000
	strong, FOR	-2.756	-12.014	6.502	1.971	-1.398	1.000
	weak, FOR	-1.171	-2.031	-0.311	0.229	-5.107	< .001
	moderate, Taper	-4.548	-13.806	4.710	1.971	-2.308	1.000
	strong, Taper	-2.448	-11.706	6.810	1.971	-1.242	1.000
	weak, Taper	-1.137	-1.997	-0.277	0.229	-4.958	< .001
moderate, SE	strong, SE	2.037	-8.407	12.482	2.223	0.917	1.000
	weak, SE	3.611	-5.647	12.869	1.971	1.832	1.000
	moderate, MS	-0.558	-1.695	0.580	0.303	-1.837	1.000
	strong, MS	1.637	-8.807	12.082	2.223	0.737	1.000
	weak, MS	3.395	-5.863	12.653	1.971	1.723	1.000
	moderate, FOR	-0.422	-1.560	0.715	0.303	-1.392	1.000

Table S.11. Post Hoc Comparisons - Status ✻ LBMa

		Mean Difference	95% CI for Mean Difference		SE	t	p holm
			Lower	Upper			
	strong, FOR	1.755	-8.689	12.199	2.223	0.789	1.000
	weak, FOR	3.339	-5.919	12.597	1.971	1.695	1.000
	moderate, Taper	-0.037	-1.175	1.100	0.303	-0.124	1.000
	strong, Taper	2.062	-8.382	12.507	2.223	0.928	1.000
	weak, Taper	3.374	-5.884	12.631	1.971	1.712	1.000
strong, SE	weak, SE	1.573	-7.685	10.831	1.971	0.798	1.000
	moderate, MS	-2.595	-13.039	7.849	2.223	-1.167	1.000
	strong, MS	-0.400	-1.538	0.738	0.303	-1.318	1.000
	weak, MS	1.358	-7.900	10.615	1.971	0.689	1.000
	moderate, FOR	-2.460	-12.904	7.984	2.223	-1.107	1.000
	strong, FOR	-0.282	-1.420	0.855	0.303	-0.931	1.000
	weak, FOR	1.302	-7.956	10.560	1.971	0.661	1.000
	moderate, Taper	-2.075	-12.519	8.369	2.223	-0.933	1.000
	strong, Taper	0.025	-1.113	1.163	0.303	0.082	1.000
	weak, Taper	1.336	-7.922	10.594	1.971	0.678	1.000
weak, SE	moderate, MS	-4.168	-13.426	5.090	1.971	-2.115	1.000
	strong, MS	-1.973	-11.231	7.285	1.971	-1.001	1.000
	weak, MS	-0.216	-1.076	0.644	0.229	-0.941	1.000
	moderate, FOR	-4.033	-13.291	5.225	1.971	-2.047	1.000
	strong, FOR	-1.856	-11.114	7.402	1.971	-0.942	1.000
	weak, FOR	-0.271	-1.131	0.589	0.229	-1.183	1.000
	moderate, Taper	-3.648	-12.906	5.610	1.971	-1.851	1.000
	strong, Taper	-1.548	-10.806	7.710	1.971	-0.786	1.000

Table S.11. Post Hoc Comparisons - Status ✻ LBMa

		Mean Difference	95% CI for Mean Difference		SE	t	p holm
			Lower	Upper			
	weak, Taper	-0.237	-1.097	0.623	0.229	-1.034	1.000
moderate, MS	strong, MS	2.195	-8.249	12.639	2.223	0.987	1.000
	weak, MS	3.952	-5.305	13.210	1.971	2.006	1.000
	moderate, FOR	0.135	-1.003	1.273	0.303	0.445	1.000
	strong, FOR	2.312	-8.132	12.757	2.223	1.040	1.000
	weak, FOR	3.897	-5.361	13.155	1.971	1.977	1.000
	moderate, Taper	0.520	-0.618	1.658	0.303	1.714	1.000
	strong, Taper	2.620	-7.824	13.064	2.223	1.179	1.000
	weak, Taper	3.931	-5.327	13.189	1.971	1.995	1.000
strong, MS	weak, MS	1.758	-7.500	11.015	1.971	0.892	1.000
	moderate, FOR	-2.060	-12.504	8.384	2.223	-0.927	1.000
	strong, FOR	0.117	-1.020	1.255	0.303	0.387	1.000
	weak, FOR	1.702	-7.556	10.960	1.971	0.864	1.000
	moderate, Taper	-1.675	-12.119	8.769	2.223	-0.753	1.000
	strong, Taper	0.425	-0.713	1.563	0.303	1.401	1.000
	weak, Taper	1.736	-7.522	10.994	1.971	0.881	1.000
weak, MS	moderate, FOR	-3.817	-13.075	5.440	1.971	-1.937	1.000
	strong, FOR	-1.640	-10.898	7.618	1.971	-0.832	1.000
	weak, FOR	-0.056	-0.916	0.804	0.229	-0.243	1.000
	moderate, Taper	-3.432	-12.690	5.825	1.971	-1.742	1.000
	strong, Taper	-1.333	-10.590	7.925	1.971	-0.676	1.000
	weak, Taper	-0.021	-0.881	0.839	0.229	-0.093	1.000
moderate, FOR	strong, FOR	2.177	-8.267	12.622	2.223	0.979	1.000

Table S.11. Post Hoc Comparisons - Status ✻ LBMa

		Mean Difference	95% CI for Mean Difference		SE	t	p_{holm}
			Lower	Upper			
	weak, FOR	3.762	-5.496	13.020	1.971	1.909	1.000
	moderate, Taper	0.385	-0.753	1.523	0.303	1.269	1.000
	strong, Taper	2.485	-7.959	12.929	2.223	1.118	1.000
	weak, Taper	3.796	-5.462	13.054	1.971	1.926	1.000
strong, FOR	weak, FOR	1.584	-7.674	10.842	1.971	0.804	1.000
	moderate, Taper	-1.792	-12.237	8.652	2.223	-0.806	1.000
	strong, Taper	0.308	-0.830	1.445	0.303	1.013	1.000
	weak, Taper	1.619	-7.639	10.876	1.971	0.821	1.000
weak, FOR	moderate, Taper	-3.377	-12.635	5.881	1.971	-1.714	1.000
	strong, Taper	-1.277	-10.535	7.981	1.971	-0.648	1.000
	weak, Taper	0.034	-0.826	0.894	0.229	0.149	1.000
moderate, Taper	strong, Taper	2.100	-8.344	12.544	2.223	0.945	1.000
	weak, Taper	3.411	-5.847	12.669	1.971	1.731	1.000
strong, Taper	weak, Taper	1.311	-7.947	10.569	1.971	0.665	1.000

Note. P-value and confidence intervals adjusted for comparing a family of 105 estimates (confidence intervals corrected using the bonferroni method).

Table S.12. Post Hoc Comparisons - Status ✻ CSA

		Mean Difference	95% CI for Mean Difference		SE	t	p_{holm}
			Lower	Upper			
moderate, Baseline	strong, Baseline	5.873	-17.883	29.628	5.219	1.125	1.000
	weak, Baseline	9.782	-11.275	30.839	4.626	2.114	1.000

Table S.12. Post Hoc Comparisons - Status ✻ CSA

		Mean Difference	95% CI for Mean Difference		SE	t	p holm
			Lower	Upper			
	moderate, SE	-0.938	-6.519	4.644	1.489	-0.630	1.000
	strong, SE	2.412	-21.343	26.168	5.219	0.462	1.000
	weak, SE	8.071	-12.987	29.128	4.626	1.744	1.000
	moderate, MS	-3.703	-9.284	1.879	1.489	-2.487	1.000
	strong, MS	2.548	-21.208	26.303	5.219	0.488	1.000
	weak, MS	6.735	-14.322	27.792	4.626	1.456	1.000
	moderate, FOR	-3.788	-9.369	1.794	1.489	-2.545	1.000
	strong, FOR	2.762	-20.993	26.518	5.219	0.529	1.000
	weak, FOR	6.836	-14.221	27.894	4.626	1.478	1.000
	moderate, Taper	-3.473	-9.054	2.109	1.489	-2.333	1.000
	strong, Taper	1.257	-22.498	25.013	5.219	0.241	1.000
	weak, Taper	5.796	-15.261	26.854	4.626	1.253	1.000
strong, Baseline	weak, Baseline	3.910	-17.148	24.967	4.626	0.845	1.000
	moderate, SE	-6.810	-30.566	16.946	5.219	-1.305	1.000
	strong, SE	-3.460	-9.042	2.122	1.489	-2.324	1.000
	weak, SE	2.198	-18.859	23.256	4.626	0.475	1.000
	moderate, MS	-9.575	-33.331	14.181	5.219	-1.835	1.000
	strong, MS	-3.325	-8.907	2.257	1.489	-2.234	1.000
	weak, MS	0.862	-20.195	21.920	4.626	0.186	1.000
	moderate, FOR	-9.660	-33.416	14.096	5.219	-1.851	1.000
	strong, FOR	-3.110	-8.692	2.472	1.489	-2.089	1.000
	weak, FOR	0.964	-20.093	22.021	4.626	0.208	1.000
	moderate, Taper	-9.345	-33.101	14.411	5.219	-1.790	1.000

Table S.12. Post Hoc Comparisons - Status ✻ CSA

		Mean Difference	95% CI for Mean Difference		SE	t	p_{holm}
			Lower	Upper			
	strong, Taper	-4.615	-10.197	0.967	1.489	-3.100	0.336
	weak, Taper	-0.076	-21.133	20.981	4.626	-0.016	1.000
weak, Baseline	moderate, SE	-10.720	-31.777	10.338	4.626	-2.317	1.000
	strong, SE	-7.370	-28.427	13.688	4.626	-1.593	1.000
	weak, SE	-1.711	-5.931	2.508	1.125	-1.521	1.000
	moderate, MS	-13.485	-34.542	7.573	4.626	-2.915	1.000
	strong, MS	-7.235	-28.292	13.823	4.626	-1.564	1.000
	weak, MS	-3.047	-7.266	1.172	1.125	-2.708	0.963
	moderate, FOR	-13.570	-34.627	7.488	4.626	-2.933	1.000
	strong, FOR	-7.020	-28.077	14.038	4.626	-1.517	1.000
	weak, FOR	-2.946	-7.165	1.274	1.125	-2.618	1.000
	moderate, Taper	-13.255	-34.312	7.803	4.626	-2.865	1.000
	strong, Taper	-8.525	-29.582	12.533	4.626	-1.843	1.000
	weak, Taper	-3.986	-8.205	0.234	1.125	-3.542	0.094
moderate, SE	strong, SE	3.350	-20.406	27.106	5.219	0.642	1.000
	weak, SE	9.008	-12.049	30.066	4.626	1.947	1.000
	moderate, MS	-2.765	-8.347	2.817	1.489	-1.858	1.000
	strong, MS	3.485	-20.271	27.241	5.219	0.668	1.000
	weak, MS	7.673	-13.385	28.730	4.626	1.658	1.000
	moderate, FOR	-2.850	-8.432	2.732	1.489	-1.915	1.000
	strong, FOR	3.700	-20.056	27.456	5.219	0.709	1.000
	weak, FOR	7.774	-13.283	28.831	4.626	1.680	1.000
	moderate, Taper	-2.535	-8.117	3.047	1.489	-1.703	1.000

Table S.12. Post Hoc Comparisons - Status ✻ CSA

			95% CI for Mean Difference				
		Mean Difference	Lower	Upper	SE	t	p holm
	strong, Taper	2.195	-21.561	25.951	5.219	0.421	1.000
	weak, Taper	6.734	-14.323	27.791	4.626	1.456	1.000
strong, SE	weak, SE	5.658	-15.399	26.716	4.626	1.223	1.000
	moderate, MS	-6.115	-29.871	17.641	5.219	-1.172	1.000
	strong, MS	0.135	-5.447	5.717	1.489	0.091	1.000
	weak, MS	4.323	-16.735	25.380	4.626	0.934	1.000
	moderate, FOR	-6.200	-29.956	17.556	5.219	-1.188	1.000
	strong, FOR	0.350	-5.232	5.932	1.489	0.235	1.000
	weak, FOR	4.424	-16.633	25.481	4.626	0.956	1.000
	moderate, Taper	-5.885	-29.641	17.871	5.219	-1.128	1.000
	strong, Taper	-1.155	-6.737	4.427	1.489	-0.776	1.000
	weak, Taper	3.384	-17.673	24.441	4.626	0.731	1.000
weak, SE	moderate, MS	-11.773	-32.831	9.284	4.626	-2.545	1.000
	strong, MS	-5.523	-26.581	15.534	4.626	-1.194	1.000
	weak, MS	-1.336	-5.555	2.884	1.125	-1.187	1.000
	moderate, FOR	-11.858	-32.916	9.199	4.626	-2.563	1.000
	strong, FOR	-5.308	-26.366	15.749	4.626	-1.147	1.000
	weak, FOR	-1.234	-5.454	2.985	1.125	-1.097	1.000
	moderate, Taper	-11.543	-32.601	9.514	4.626	-2.495	1.000
	strong, Taper	-6.813	-27.871	14.244	4.626	-1.473	1.000
	weak, Taper	-2.274	-6.494	1.945	1.125	-2.021	1.000
moderate, MS	strong, MS	6.250	-17.506	30.006	5.219	1.197	1.000
	weak, MS	10.438	-10.620	31.495	4.626	2.256	1.000

Table S.12. Post Hoc Comparisons - Status ✻ CSA

		Mean Difference	95% CI for Mean Difference		SE	t	p holm
			Lower	Upper			
	moderate, FOR	-0.085	-5.667	5.497	1.489	-0.057	1.000
	strong, FOR	6.465	-17.291	30.221	5.219	1.239	1.000
	weak, FOR	10.539	-10.518	31.596	4.626	2.278	1.000
	moderate, Taper	0.230	-5.352	5.812	1.489	0.155	1.000
	strong, Taper	4.960	-18.796	28.716	5.219	0.950	1.000
	weak, Taper	9.499	-11.558	30.556	4.626	2.053	1.000
strong, MS	weak, MS	4.188	-16.870	25.245	4.626	0.905	1.000
	moderate, FOR	-6.335	-30.091	17.421	5.219	-1.214	1.000
	strong, FOR	0.215	-5.367	5.797	1.489	0.144	1.000
	weak, FOR	4.289	-16.768	25.346	4.626	0.927	1.000
	moderate, Taper	-6.020	-29.776	17.736	5.219	-1.153	1.000
	strong, Taper	-1.290	-6.872	4.292	1.489	-0.867	1.000
	weak, Taper	3.249	-17.808	24.306	4.626	0.702	1.000
weak, MS	moderate, FOR	-10.523	-31.580	10.535	4.626	-2.274	1.000
	strong, FOR	-3.973	-25.030	17.085	4.626	-0.859	1.000
	weak, FOR	0.101	-4.118	4.321	1.125	0.090	1.000
	moderate, Taper	-10.207	-31.265	10.850	4.626	-2.206	1.000
	strong, Taper	-5.478	-26.535	15.580	4.626	-1.184	1.000
	weak, Taper	-0.939	-5.158	3.281	1.125	-0.834	1.000
moderate, FOR	strong, FOR	6.550	-17.206	30.306	5.219	1.255	1.000
	weak, FOR	10.624	-10.433	31.681	4.626	2.296	1.000
	moderate, Taper	0.315	-5.267	5.897	1.489	0.212	1.000
	strong, Taper	5.045	-18.711	28.801	5.219	0.967	1.000

Table S.12. Post Hoc Comparisons - Status ✻ CSA

		Mean Difference	95% CI for Mean Difference		SE	t	p holm
			Lower	Upper			
	weak, Taper	9.584	-11.473	30.641	4.626	2.072	1.000
strong, FOR	weak, FOR	4.074	-16.983	25.131	4.626	0.881	1.000
	moderate, Taper	-6.235	-29.991	17.521	5.219	-1.195	1.000
	strong, Taper	-1.505	-7.087	4.077	1.489	-1.011	1.000
	weak, Taper	3.034	-18.023	24.091	4.626	0.656	1.000
weak, FOR	moderate, Taper	-10.309	-31.366	10.748	4.626	-2.228	1.000
	strong, Taper	-5.579	-26.636	15.478	4.626	-1.206	1.000
	weak, Taper	-1.040	-5.259	3.179	1.125	-0.924	1.000
moderate, Taper	strong, Taper	4.730	-19.026	28.486	5.219	0.906	1.000
	weak, Taper	9.269	-11.788	30.326	4.626	2.003	1.000
strong, Taper	weak, Taper	4.539	-16.518	25.596	4.626	0.981	1.000

Note. P-value and confidence intervals adjusted for comparing a family of 105 estimates (confidence intervals corrected using the bonferroni method).
